# Children Exposed to Intimate Partner Violence: Association Among Battered Mothers’ Parenting Competences and Children’s Behavior

**DOI:** 10.3390/ijerph17041134

**Published:** 2020-02-11

**Authors:** Ana Rosser-Limiñana, Raquel Suriá-Martínez, Miguel Ángel Mateo Pérez

**Affiliations:** 1 Department of Communication and Social Psychology, University of Alicante, San Vicente del Raspeig, 03690 Alicante, Spain; raquel.suria@ua.es; 2 Department of Social Work and Social Services, University of Alicante, San Vicente del Raspeig, 03690 Alicante, Spain; ma.mateo@ua.es

**Keywords:** intimate partner violence (IPV), parenting, children, mother–child interactions, behavioral problems: Child Behavior Checklist (CBCL), shelters

## Abstract

Background: Exposure to violence perpetrated on a mother by her intimate partner (IPV or intimate partner violence) has an impact on the psychosocial adjustment of her children. In addition, the violence suffered by mothers could affect parental competences. Methods: Through the Child Behavior Checklist (CBCL), this work analyzes the psychosocial adjustment in children between 6 and 17 years old who live with their mothers in shelters after having experienced IPV situations. It also explores the association between mothers’ parenting competences and children’s adjustment in shelters. Results: The evaluation shows a negative correlation between the quality of mothers’ care of their children during their stay in shelters and the rate of children’s behavioral problems, so that the better the parental competences of mothers, the lower the rate of behavioral problems presented by children. Conclusions: As a result of IPV, mother–child relationships can be affected. Children exposed to IPV may exhibit more externalizing behavior problems and their mothers may have difficulty demonstrating competent parenting behaviors while living in a shelter. Work should be aimed at reestablishing parenting competences in mothers and the quality of mother–child interactions while they remain in the shelters, in an effort to mitigate the psychosocial consequences of IPV for their children.

## 1. Introduction

### 1.1. Violence Experienced by Children in the Context of IPV

Intimate partner violence (IPV) is one of the most common forms of violence against women and includes physical, sexual, and emotional abuse and controlling behaviors by an intimate partner.

Many women who experience violence from an intimate partner are also mothers. This is the case for 73% of women who experience IPV [1]. Children experience IPV in their homes through all of their senses and so become more than just witnesses [2].

In contexts of IPV against mothers, their children see, hear, and are involved in these episodes of violence [3]. Children may not always observe the violence directly (since, in many instances, the abuse is manifested as psychological and controlling behavior by the perpetrator), but they are still aware of the abuse that is taking place [2,4]. Additionally, fathers often use children to control and hurt their mother, weakening her maternal authority [5,6].

Consequently, it is important to not only focus on the problem of IPV against women, but also pay attention to the situation of their children, given that numerous organizations consider this to be a far-reaching problem, with 275 million children around the world exposed to violence at home [7].

In Spain, the 2015 official macro-survey on gender- based violence [8], carried out with a sample of 9807 resident Spanish women who had ever suffered from IPV, indicates that 63.6% of their children had witnessed situations of violence at some point, and 64.2% of the children had experienced violence themselves.

In the particular case of Spain’s Valencian Community [9], in a study on the situation of women living in shelters (p. 149), 72.0% came with their children. According to this report (p. 166), 62.9% of the women reported that their children had been eye-witnesses of abuse, 66.1% had heard it, and 30.6% had suffered from it directly.

These experiences of IPV at home can affect children in a negative way and can have a clear impact on their psychosocial adjustment [4]. In fact, during recent decades, an important body of research has developed about children’s exposure to adult IPV at home (direct involvement, as direct eyewitnesses, and indirect exposure), revealing that these children can display symptoms of post-traumatic stress, a lack of social skills, and emotional and behavioral problems [10,11,12,13]. 

More recent studies have shown similar outcomes, presenting a prevalence of depressive symptoms, insecurity, and post-traumatic stress disorder (75.8%), followed by behavioral problems and aggression (32.6%), and declining academic performance and bullying (20%) [14], or concluding that exposure to IPV was positively associated with child adjustment problems for externalizing and internalizing behaviors [15,16]. IPV is a form of child maltreatment, with harmful and long-term consequences for health and well-being [17,18]. 

According to the Project on Human Development in Chicago Neighborhoods (PHDCN) [19], the association between IPV and child behavioral problems may attenuate as the age of the child at the time of exposure increases. Furthermore, when IPV exposure is assessed, the child’s age moderates the association between IPV exposure and externalizing problems [20]. In fact, the relation is stronger when IPV exposure is assessed in younger children. The length of time over which children are exposed to IPV could be as important as age.

Studies that focus on analyzing the influence of sex on the effects of exposure to IPV in children have not been conclusive. While some studies consider that there is a greater risk of presenting externalizing behavior (hostility and aggression) in boys [12], or a greater risk of internalizing behavior (self-blame and shame) in girls [21], others indicate that these differences are not significant [22] or not observed [23,24].

Furthermore, children’s emotional and behavioral problems will have an impact on other relevant areas of their lives, such as school achievement [25,26,27].

In addition, it is well-known that the quality of the relationship between parents and children is a predictor of a child’s future development and numerous studies have shown that a positive association with an adult caregiver can serve as a protective factor that mitigates the effects of adverse circumstances [13,28]. In situations of IPV, the quality of child-rearing and the emotional bond also moderates the result of evaluations of children’s psychosocial adjustment [29,30].

The effects of abuse on a woman, such as raised stress levels, emotional problems, financial precariousness, job insecurity, and so on, can also affect how she carries out her maternal role, making it difficult for her to meet the needs of her children [31,32,33,34]. They can also cause deficits in her affective responses, as well as difficulties in carrying out parenting tasks [29,30,32]. 

Likewise, there is research which suggests that, in homes affected by IPV, there is a greater propensity for severe disciplinary action and aversive interactions with children [33,35,36]. Mothers often face difficulties in establishing limits for their children, resulting in a situation where children are overprotected or, in contrast, situations of negligence and abandonment [32].

However, other researchers have found that victims of IPV may display compensatory behaviors in their interactions with their children, in particular, positive discipline, affection, and consistent parenting styles [37], because they mediate the association between this situation and children’s problems, so that when a mother shows a caring attitude towards her children, they develop a greater capacity for self-regulation and effortful control than when she uses an intrusive and harsh style [38].

The importance of the family environment, and more specifically, the type of parent–child interaction, affects the adjustment of adolescents in a very special way [39]. Recent research has shown that a poorer personal well-being and social well-being were associated with less parental warmth [40]. In fact, some research has shown that indulgent parenting, characterized by the use of reasoning and warmth practices, can act as a protective factor [41]. 

The accumulation of stressful life events has been shown to be an important trigger of psychological maladjustment in adolescents [39]. For instance, experiences in contexts of IPV can influence the presence of disruptive behavior in adolescents and the assumption of roles of dominance/submission at home [42]. For example, a longitudinal study carried out over the course of 20 years with 543 subjects [42] showed that after behavioral disorders in adolescence, exposure to violence between parents was the principal risk factor for the perpetuation of violence against an intimate partner in adulthood. This association has been reflected in further research. Some studies have found that witnessing IPV is significantly associated with the perpetration of victimization, physical aggression, and injury [43,44].

### 1.2. The Present Study

The aim of this study is to better understand the association between IPV against mothers and the presence of emotional and behavioral problems in their children. This article explores the variables that might act as moderating factors in the impact of exposure to violence among children. In fact, not all children show problems after exposure to IPV perpetrated against their mothers [45].

With prior research as a point of departure, this work presents the following specific objectives:

1. To explore the existence of differences between children exposed to IPV and their peers in the general population in terms of behavioral problems;
2. To describe the parenting competences of mother–child interactions in the context of social care for women who are victims of gender-based violence;
3. To explore whether there is an association between behavioral problems in children and parenting competences in mother–child interactions in the shelters.

The hypotheses of the study are as follows:

1. Children who have experienced gender-based violence against their mothers have a higher rate of behavioral problems;
2. Women who are victims of gender-based violence may have reduced parenting competences;
3. There is an association between the quality of parenting competences demonstrated by mothers while living with their children in shelters and the rate of child behavioral problems.

It is important to note that, although there is a wide body of international research on the topic of children exposed to violence perpetrated against their mothers by an intimate partner, in Spain, where the present study has been carried out, this has only been a topic of interest for the past few years, and there is less literature in this area based on Spanish population groups. There are even fewer studies that compare results with the normative population or that highlight intervening factors, such as the children’s age or sex or the parenting behaviors of the mothers. This delay is probably because the first law against gender-based violence in Spain was not adopted until 2004.

## 2. Materials and Methods 

### 2.1. Participants

We carried out a transversal descriptive study of children and their mothers in shelters that specialize in assisting victims of gender-based violence in the Valencian Community (Spain). All of them were cared for in shelters for a year.

Residential care services linked to the Valencian network for social services for victims of gender-based violence and their children provide a series of centers that offer shelter for local and foreign women who have been victims of physical and/or psychological abuse and their children. These centers serve as a resource for comprehensive care whenever the situation might require it, due to its gravity in terms of the abuse suffered, the lack of an alternative shelter, or when women lack financial means to confront the abusive situations and there are risks to their physical and/or psychological health. In the Valencian Community (Spain), these full-service residential care centers include an urgent care shelter (city of Alicante, for 12 women), three full recovery centers (city of Alicante, for 27 women; city of Castellón, for 35 women; and city of Valencia, for 24 women), and some sheltered housing. Victims of IPV can stay in centers for a maximum of one year. After this, they receive support to find work and housing and their situation is monitored by social services.

The inclusion criterion for this study was children aged 6 or older, given that the CBCL instrument has been validated for this age range in Spain.

During the study period, 50 children (58.8%) aged 6 or older stayed in shelters in the Valencian Community (Spain). After discarding those whose mothers did not give their consent to the study or did not speak Spanish, 46 children aged between 6 and 17 and living with their mothers (*N* = 29) in shelters for victims of IPV were included in the study, representing 92% of the children aged 6 or older who were looked after in these centers during the year. Regarding sex, 52.2% were girls and 47.8% were boys, with an average age of 11 years (*M* = 11.15; *SD* = 2.60).

The majority of the mothers had completed elementary school (65.5%). Of these women, 96.6% had suffered from physical violence, 93.1% psychological violence, and 41.4% sexual violence.

### 2.2. Measures

#### 2.2.1. Demographic Data Collection Sheet

For the purpose of examining the profiles of children and their mothers.

#### 2.2.2. Child Behavior Checklist (CBCL)

The CBCL is a behavioral inventory ) [46,47] frequently used for studying behavioral problems in children of women who are victims of gender-based violence [23,48] and it allows the evaluation of eight first-order syndromes or narrowband syndromes (aggressive behavior, attention problems, social problems, thought problems, somatic complaints, being withdrawn, and anxiety/depression). It has been demonstrated that the instrument has adequate psychometric properties [47]. Through an exploratory factor analysis, three factors were obtained, explaining between 59% and 88% of the variance between items that were part of the scale, in different samples classified by age and sex: an internalizing factor (withdrawal, somatic complaints, and anxiety/depression), an externalizing factor (rule breaking and aggressive behavior), and a mixed factor that includes different social and attention problems. In addition, the reliability calculated using Cronbach’s alpha index in original studies was α = 0.78 and α = 0.97. In this study, we used Spanish-validated scales [49]. We did not require a control group because the comparative element was the normative group. The normative sample comprised 1430 children aged 6 to 17 (50% boys and 50% girls) from schools representing high, medium, and low socioeconomic levels in three Spanish cities: Barcelona, Madrid, and Reus (Tarragona). In this case, the factor analysis confirmed the structure of this instrument, with an excellent reliability and exactitude, and Cronbach’s alpha levels of over .80.

#### 2.2.3. Scale of Competent Parenting Behavior 

This instrument was designed specifically for this study, based on the definition of parenting competences provided by Spanish studies [50,51]. The scale has five yes/no items that are related to aspects of parenting by the mother, involvement in daily life, affection, and discipline To dichotomize the scores, a global score was calculated and a median-based cut-off point was established [52,53].

The evaluation of the quality of parenting competences in mother–child relationships is based on the parenting competence concept [50]:

*“The group of capabilities that allows parents to address the work of being parents in a flexible and adaptable way, according to the evolutionary and educational needs of their children, by standards considered acceptable to society, and taking advantage of all opportunities and support provided by systems influencing the family in order develop these skills.”*

(p. 115)

For these authors, parental competences are multidimensional because they combine cognition, affect, behavior, and communication; bidirectional, because they promote the personal and social adjustment of individuals and allow them to identify what context they can provide; and dynamic, because they must be adapted to the moment that people live in and their abilities and capacities.

These competencies—cognition, affect, behavior, and communication—have been adapted to the possibilities of institutional life.

### 2.3. Procedure

In order to begin the study, we obtained authorization from the public entity responsible for shelters for gender-based violence victims. 

After being informed of the research objectives, mothers gave their oral consent to participate in the study, and to assess the children’s behaviors. In addition, they filled-in the parental report version of the Child Behavior Checklist (CBCL). 

By indication of the public entity, in order not to hinder the daily life of the shelters, the staff who were directly responsible for attending to the women at the shelters cooperated with the study, filling-in the scale of competent parenting behavior, instead of the research team.

The staff had received qualified training because they were psychologists, social workers, and educators with experience in intervening with battered women in shelters. In addition, they received instructions from the researchers during several training sessions on what behaviors to observe and how to record them. The research design did not include assessments of the degree of agreement between the professionals who applied the questionnaire and the trainers. Given the previous level of training and the small group work, the authors considered that the contents and indications when applying the questionnaire had been clearly and precisely understood by the staff. In order to avoid biases in the evaluation by the staff, they were able to maintain direct contact with the research team in person and/or by telephone, which allowed them to resolve all doubts and incidents that arose in the process.

Staff answered the scale on the basis of their knowledge of mothers and their habitual behavior, but without subjecting them to an examination situation.

As the sample included mothers with multiple children with them in the shelter, mothers filled-in the CBCL with respect to each child independently and professionals evaluated the mother–child relationship with respect to each individual child.

Sociodemographic data were obtained from the reports collected in the case files. 

We complied with the requirements of the Ethics Committee of the University of Alicante (Spain) and the ethical principles of the Declaration of Helsinki (October 2000) for research with human subjects. Confidentiality of all information was guaranteed by encrypting the files.

### 2.4. Statistical Analyses 

#### 2.4.1. Child Behavior Check List (CBCL) 

With regards to the first research objective, after a descriptive analysis (frequencies and percentages), a comparison of averages was carried out using Student’s *t* test, as well as an analysis of the possible relationships between variables. In order to counter the sample size, tests of statistical significance were accompanied by a corresponding calculation and interpretation of the effect of size, using Cohen’s *d* (typified difference of the average), which indicates whether the magnitude of differences is small, moderate, or large.

#### 2.4.2. Scale of Competent Parenting Behavior

For construction of the scale, and after reviewing previous studies [50,51], a list of 10 items containing possible parental competencies was initially obtained. To this end [52], different criteria were established to generate the items: they should be easy for the participants to understand, they should include closed-ended questions, and the number of items should be doubled in order to discard those that were least suited to the objective of the scale. This list was analyzed using the Delphi technique of consensus by different experts (psychologists and social workers of the Valencian government). After the first round, a list of eight questions was obtained, with Likert-type answers of five points (1 = no agreement; 2 = little agreement; 3 = some agreement; 4 = much agreement; 5 = totally agree), aimed at obtaining the competencies of mothers towards their children.

Five of these items concerned mothers’ behavior towards their children and three concerned children’s behavior towards their mothers. For this reason, it was finally decided to use only the five items exclusively referring to maternal behavior. These items were dichotomized in order to facilitate the analysis. For this purpose, the same criterion was established, consisting of reducing the number of alternatives for each item in order to eliminate possible biases, transforming the alternatives into two options (yes/no). The binomial model was used since it allows us to find out if a variable adequately fits a probability model. This statistical contrast was used because it is a nonparametric test used when the sample size is small and the variables are dichotomous. The results were adjusted to the model (*p* > 0.05).

In order to meet the second research objective, the frequencies and percentages obtained for the different competences in mothers’ items on the scale were also analyzed. We used the chi-squared test to determine whether there were differences in the mothers’ competence items based on the sex or age of the children. Before doing this, we categorized the age variable into different groups.

Finally, to explore the third research objective, the possible association between children’s behavioral problems and the parenting competences, Spearman’s correlation coefficient was used, given that the sample distribution was not a normal distribution.

The confidence level used to determine statistical significance in all of the analyses was 95%. 

## 3. Results

### 3.1. Sociodemographic Characteristics of the Children and Their Mothers

Table 1 presents the sociodemographic characteristics of the children and their mothers, extracted from their case file.

### 3.2. Child Behavioral Problems

**Figure 1:**
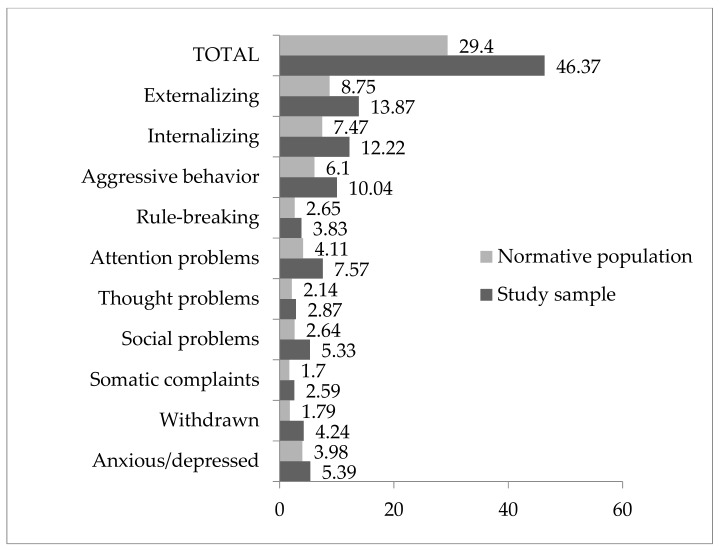
Difference of means with a normative population (direct scores).

In relation to the first objective (see Figure 1), the comparison of averages indicated statistically significant differences between the sample studied and normative population, with higher scores in the sample studied regarding anxiety (*t* = 6.65, *p* = 0.048, *d* = 0.34), being withdrawn (*t* = 2.46, *p* < 0.001, *d* = 0.86), social problems (*t* = 2.96, *p* < 0.001, *d* = 0.86), attention problems (*t* = 3.376, *p* < 0.001, *d* = 0.85), and aggressiveness (*t* = 4.096, *p* = 0.002, *d* = 0.59). There were also significant differences in the broad band syndromes of internalization (*t* = 4.145, *p* = 0.003, *d* = 0.57) and externalization (*t* = 4.418, *p* = 0.002, *d* = .58) and in the total behavioral problems (*t* = 4.465, *p* < 0.001, *d* = 0.66). Considering that effect sizes of less than 0.20 are considered small, from 0.2 to 0.8 are considered medium, and more than 0.8 are considered large, the study of the effect size indicates that the magnitude of these differences is greater for withdrawal/depressive and attention problems.

Furthermore, between 56% and 89% of children were in a normal range, between 2% and 13% were in the limit range, and between 8% and 37% were in the clinical range, depending on the syndromes. In particular, 17.4% were in the clinical range for the total behavioral problems, 19.6% were in the clinical range for internalizing behaviors, and 17.4% were in the clinical range for externalizing behaviors.

In the analysis of the narrow band syndromes, we observed a greater presence of attention problems (37%), being withdrawn (28.3%), social problems (19.6%), and rule-breaking behavior (15.2%) in the clinical range.

### 3.3. Competent Parenting Behavior 

The results for the second study objective illustrate the difficulties some mothers have in meeting the needs of their children (Table 2).

This was revealed in terms of a lack of shared playtime (item 1: *n* = 7; 15.9%), limited displays of affection (item 2: *n* = 11; 24.4%), and a moderate level of enjoyment during the time that mothers spend with their children (item 5: *n* = 23; 51.1%). They also showed difficulties in establishing adequate norms and boundaries (item 3: *n* = 9; 20%) and in some activities, were demanding or overprotective and did not sufficiently stimulate their children’s autonomy (item 4: *n* = 9; 20%).

Regarding the parenting competences exhibited by mothers in their relationships with their children by sex, the analyses do not show statistically significant differences (*p* = 0.395). The age of the children was also not relevant (*p* = 0.312).

### 3.4. Association between Behavioral Problems in Children (CBCL) and Mothers’ Parenting Competences

An examination of the association between children’s behavioral problems and the parenting competence scale (see Table 3) showed a negative association. 

With regard to the possible association between behavioral problems in children and parental competences in mothers, correlation indices indicated an association between some competences with the CBCL full scale, as well as between some of the items. Therefore, when examining the most representative items of these associations, an association was observed between the item “Mother plays with him in the center, they spend time together” and social problems (r = −0.431; *p* > 0.01), and between the item “Mother enjoys sharing her time with her child” and attention problems (r = −0.311; *p* > 0.05), disruptive behaviors (r = −0.406; *p* > 0.01), aggressiveness (r = −0.394; *p* > 0.01), and externalizing behaviors (r = −0.425; *p* > 0.05). Likewise, the competence “Mother stimulates the child’s personal autonomy” was associated with anxiety problems (r = −0.330; *p* > 0.01), attention problems (r = −0.380; *p* > 0.01), disruptive behaviors (r = −0.386; *p* > 0.01), aggressiveness (r = −0.381; *p* > 0.01), and externalizing behaviors (r = −0.408; *p* > 0.05). In all cases, an inverse correlation was observed, so the better the parental competences of mothers, the lower the rate of behavioral problems presented by children.

## 4. Discussion

The data offered by official Spanish sources on the number of children involved in situations of IPV with their mothers [8] indicate that this is a far-reaching problem that requires attention in terms of both research and intervention. The situation is similar in the Valencian Community, where in 72% of cases of women supported in shelters, their children have also suffered from violence in different ways [9].

Firstly, this study shows the profile of victims of IPV in shelters; women who have suffered physical, psychological and sometimes sexual abuse, and their children.

On the other hand, the research shows that exposure to IPV is often positively associated with child adjustment problems [4]. The results of this study show a greater presence of behavioral and emotional problems among children who have been exposed to IPV. In comparison with the normative population, their average scores are higher for all of the syndromes [13,19,23,51,54]. These differences are statistically significant in all of the narrow band syndromes, with the exception of “somatic complaints” and “thought problems.” The differences are also significant in the larger syndromes of internalization, externalization, and total behavioral problems. There is a slightly higher percentage of clinical cases in terms of internalizing behaviors compared to externalizing behaviors and compared to total behavioral problems. This result differs from other works in which the presence of externalizing behaviors is greater [51,55]. This result could be because this study was carried out with an institutionalized population, so thus took place in a setting with a greater control of externalizing behaviors.

The effect size—moderate or elevated—confirms the magnitude of differences between the sample and normative population scores. The effect size is moderate for aggressive behavior and internationalization and for externalization and total behavioral problems, and elevated in the dimension of “being withdrawn” and “social problems” (*d* = 0.86) and “attention problems” (*d = 0*.85), which confirms the differences with the normative population and highlights that it is precisely in terms of these two syndrome scales that the differences are greater.

As other authors have indicated [10], not all of the children show behavioral problems. For this reason, it is necessary to consider in more depth the types of factor that can increase the risk.

One of these factors could be the mother–child relationships. Contrary to what is expressed by some authors [37], the results show that women who arrive at shelters with their children after suffering from violence inflicted by their partners can face difficulties in being involved in the activities of their children’s everyday lives and/or present difficulties in their parenting competences [32]. It is possible that high levels of stress and psychological problems derived from the experience of violence, such as anxiety, depressive symptoms, lowered self-esteem, and post-traumatic stress disorders [34,56], could have negative effects on these women fully carrying out their parenting roles or could prompt them to delegate the care of their children to professionals or other workers at the center. Furthermore, living with a partner who assaults, insults, or disparages a mother in front of her children could also have diminished her ability to carry out her maternal role or to establish norms and boundaries for her children [37].

The evaluation of the possible connection between mother–child relationships and children’s adjustment shows that mothers who do not pay much attention to their children while living with them in the shelter (as judged by staff) tend to have children with greater behavioral problems—in this case, externalizing behaviors (aggressiveness and disruptive conduct) and attention problems [50,51,55,56,57]. 

Parenting behaviors mediate the effect of exposure to gender-based violence in children; when a mother shows a caring attitude towards her children, they develop a greater capacity for self-regulation and modulation of their conduct [36,58]. Probably, maternal attention becomes a containing factor for impulsivity and nervousness in children, diminishing the frequency of inappropriate behavior (aggressiveness and disruptive conduct). However, in situations of IPV in which, as shown above, mothers are subjected to high levels of stress and where there has been a decrease in their involvement in child-rearing, these mechanisms of affection and containment can decline and children can display more problems of externalizing behaviors [59,60]. In addition, the environments in shelters, where residents live following highly traumatic circumstances, can make positive parenting practices difficult.

## 5. Conclusions and Proposals for Social Intervention 

Exposure to IPV leaves a mark on children that often entails a psychosocial imbalance as a consequence of the need to adapt to and overcome these situations. Intervention to protect them often entails the need to leave home, school, and friends, which also affects their psychological well-being and can lead to problematic behavior.

The first conclusion that can be obtained from these observations, is that, once again, IPV has shown negative effects both in children and the mother–child relationship, and there is a need to explore ways of reducing the behavioral problems in children and improving the quality of child-rearing [61,62,63].

As a consequence, the second conclusion is that the time children spend with their mothers in shelters should be used to detect and address the difficulties of these children [64,65], but it should also serve to reestablish mothers’ psychosocial adjustment and mothers’ competences, not just for the sake of their well-being and to empower them to regain control over their lives, but also because, by regaining these competences, their children’s recovery from the repercussions of violence may be easier.

To achieve these goals, it is necessary to consider several recommendations.

Firstly, children must be evaluated based on several determinant variables before the intervention: characteristics of the exposure; the effects of exposure to violence on the child’s mental health and adaptation; and mediating variables of the child, the family, and the social context [66]. The type of therapeutic intervention should be based on the type of sequelae detected, the level of child’s development, and the family context. Therefore, interventions with smaller children usually incorporate games, while interventions with adolescents are based on treatments originally aimed at adults, but take into account the assumption of risks and social pressures, which are characteristics of adolescence. Some treatments focus on the specific adaptation problems related to exposure to violence (for example, aggression or behavior related to exposure to abuse), while others involve preventive strategies for dealing with the risks that will take place in violent families. Other treatments are designed to help children and families in specific transitions, for example, when the mother and children leave home to go to a shelter for women who have experienced IPV or when they are going to leave the shelter.

In addition, interventions can be carried out in the form of the treatment of traumatic sequelae at an individual level, in psychoeducational programs and support groups, or in intervention programs for children and their mothers [67]. Individual treatments allow personalized attention to traumatic signals, distorted thoughts, and specific behavioral interactions. In group treatments, the general objectives are to deal with beliefs and attitudes about violence, manage reactions to violence, and develop problem-solving skills.

Regarding the intervention with mothers, it should provide instrumental and emotional support during their stay in the shelter, increase their acknowledgement that their children have also been affected by their IPV experience, and teach them strategies for developing an effective educational style. These programs are aimed at improving communication skills and management and care strategies for the children. They can be individual- or group-based [68]. 

Multileveled programs of mothers and children working both separately and together across sessions might generate the most successful psychosocial recovery for mothers and children who have experienced violence in the home [69]. However, given the heterogeneity of existing interventions and the limitations of current research [70], it is not yet clear which interventions or intervention components are most effective in addressing the needs of women parenting in the context of IPV, so further research is needed.

### Limitations of the Study

This study has several limitations. Some of them are related to the sample size. This is because the study is focused on a very specific group (children institutionalized with their mothers in shelters) with restrictive conditions to access to protect them. For this reason, the research does not have a control group. The age range of the Spanish scales (6–17 years) in the CBCL instrument used also affected the sample size. However, the sample represents 92% of this group. 

Another limitation could be that, due to the distinctiveness of the study subjects and the challenges of accessing this population, data about mother–child relationships were collected through the shelters’ staff and not directly through researchers, to ensure the safety and anonymity of the study subjects [71]. Although the research team gave the staff prior training, this circumstance could affect the evaluation of the study subjects. Therefore, in future research, we hope to find greater easiness through the authorities. 

Likewise, it should be noted that the scale of parental skills adapted to the specificity of the studied group has been used. In subsequent studies, a validation of the applied scale would be necessary, in order to guarantee validity in all its dimensions.

On the other hand, there is a possibility that parenting behaviors are different in a non-home environment and may not represent the parenting style used previously. However, the stay in the shelters does not last for more than one year, which prevents longitudinal studies.

In addition, it is possible that mothers’ parenting and children’s symptoms may both be affected by the shelter setting. As a consequence, it is necessary to compare these results with non-institutionalized groups in future analyses.

The limitations of the research condition the generalization of the data to all women victims of gender violence and their children. This is an exploratory study, so the results point to the cases studied and only indicate trends. Overcoming the limitations of this work in future research is a firm purpose of the authors.

## Figures and Tables

**Table 1:** Sociodemographic characteristics of the sample.

**Children**	**Age**		**Range**	**Average; *SD***
			6–17	10.96; 2.80
	**Sex**		n	%
		- Boy	22	47.80; 52.20
		- Girl	24	
**Women **(n = 29)	**Age**		**Range**	**Average; *SD***
			27–48	36; 5.70
	**Education**		**n**	%
		- Elementary	19	65.5
		- Secondary/professional training	9	31
		- Higher	1	3.4
	**Violence suffered**		**n**	**%**
		- Physical	28	96.60
		- Psychological	27	93.10
		- Sexual	12 (*)	41.40
	**Nº children in the shelter**		**n**	**%**
		- 1	16	55.17
		- 2	9	31.03
		- 3	4	13.79

(*) All women who had suffered from sexual abuse had also experienced physical and psychological abuse.

**Table 2:** Frequency and percentages for the items of the scale related to parenting competences.

Items of the Scale related to Quality of Mother Parenting Competences	No		Yes	
	n	%	n	%
1. Mother plays with him/her in the center—they spend time together	37	84.1	7	15.9
2. Mother is affectionate with child’s demands	34	75.6	11	24.4
3. Mother establishes rules and limits	36	80.0	9	20.0
4. The mother stimulates the autonomy of the child	24	54.5	20	45.5
5. The mother enjoys the time she spends with her son/daughter	22	48.9	23	51.1

These items were developed based on the definition of parenting competences [47].

**Table 3:** Significant relationship between behavioral problems of children and parenting competences in mothers.

	Mother Plays with Him in the Center, They Spend Time Together	Mother is Affectionate with Child’s Demands	Mother Sets Appropriate Rules and Limits	Mother Enjoys Sharing Her Time with Her Child	Mother Stimulates the Child’s Personal Autonomy
Anxiety/depression	−0,247	−0,025	−0,001	−0,243	−0,330 ^*^
Withdrawn	−0,243	0,057	0,032	**−0,087**	0,117
Somatic complaints	−0,086	−0,077	−0,095	−0,148	−0,167
Social problems	−0,431 ^**^	0,032	0,090	−0,271	−0,250
Thought problems	−0,102	0,039	0,054	−0,109	−0,175
Attention problems	−0,296	−0,062	−0,139	−0,413 ^**^	−0,380 ^*^
Rule-breaking behaviors	−0,190	−0,311 ^*^	−0,315 ^*^	−0,406 ^**^	−0,386 ^*^
Aggressiveness	−0,238	−0,289	−0,269	−0,394 ^*^	−0,381^*^
Internalizing behaviors	−0,228	−0,018	−0,022	−0,192	−0,165
Externalizing behaviors	−0,240	−0,315 ^*^	−0,301	−0,425 ^**^	−0,408 ^**^
Total	−0,283	−0,128	−0,123	**−0,329**	−0,379 ^*^

Significant correlations in bold. * significant at *p* < 0.05; ** significant at *p* < 0.01.
